# Editorial and Miscellaneous

**Published:** 1866-07

**Authors:** H. Marshall

**Affiliations:** Dentist


*Editors Atlanta Medical and Surgical Journal:*

The science of medicine, which has made rapid advances in the last half century, is receiving that attention from you and your coadjutors, that its importance demands.

There is no study upon which more constant thought, and laborious and scrutinizing observation ought to be earnestly bestowed. The life and health of the community, to a very considerable extent, are in the hands of the practitioner of medicine; he ought, therefore, to be a thoroughly educated man in this, decidedly the most important of the learned professions. To promote this is doubtless the grand object of your valuable Journal.

It is not my place or intention to write a treatise upon the science of medicine. There is a subject intimately connected with it, however, upon which a few reflections may be acceptable to your readers.

The eruption and management of children's teeth, as a general thing, does not receive that attention which its importance demands, and although we have many journals devoted to Dental

Science, publishing elaborate articles upon the management of the teeth, diffusing light upon this subject; multitudes remain in ignorance in reference to the baneful influence that neglected teeth, especially in young children, may have upon the general health.

First and second dentition are subjects rarely considered by those who are most deeply interested in them, and yet there is nothing of greater importance, or that will contribute more to the health and well-being of children, present and future, than timely and judicious attention to the development and care of the teeth. A vast amount of suffering could be saved, and even life itself, if this branch of dental science was properly understood and appreciated.

From some cause, an idea very generally prevails that when the dentist has extracted an aching tooth, filled with gold those that are decayed, or has inserted artificial fixtures to supply the loss of the natural organs, that his work is at an end—his mission as a benefactor to the race complete. This is, however, a most pernicious and fatal mistake. We say pernicious, because it is untrue; it is a libel upon the profession, and fatal because thousands of teeth are yearly sacrificed, on account of unpardonable oversight on the part of those whose duty it is to impart correct information upon this neglected topic. It is true there are men in the pofession, whose aspirations lead them to nothing higher, and follow it simply for the *lucre* connected with it. To this there are many honorable exceptions.

Our Dental Colleges are turning out men of rare genius, who have had every opportunity for acquiring a thorough knowledge of the profession, and who devote their time and talents to the same, in all its branches, especially the management of children's teeth. This knowledge is not always confined to the graduates of a Dental College. There are many men in the profession who commenced practice before such an institution was ever established, as well as many who have never enjoyed the facilities of such an institution, who are, nevertheless, masters of their business.

With a little discrimination it will be an easy matter to find some man who understands this department of Dental Surgery—the management of children's teeth—and when found, no time should be lost in having their teeth examined. If they are sound and in good condition, the examination will cost comparatively nothing; but should a temporary tooth be decayed or otherwise diseased, by all means have this damage repaired before it becomes painful. We fill many teeth for children from three years old and upward. Every medical man knows that an inflamed and aching tooth in the mouth of a child, often seriously disturbs the great life forces and produces the most serious consequences. Should the child be of firm and robust constitution, and the health not impaired from this cause, don't think for a moment that it will escape without serious trouble from another greater.

The child, at birth, has in its mouth the germs of fifty-two teeth. These are in a soft and pulpy state, and easily injured.

When the first or temporary teeth are fully developed there are ten in each jaw; these should be very tenderly cared for to prevent disease from being communicated to the soft and pulpy germs of the permanent ones just beneath them. Properly treated, these temporary teeth will remain in the mouth of the infant until the fangs are absorbed, and nature, by this beautiful operation, throws them out of the system, and the permanent teeth begin to make their appearance, which will usually be between the fifth and sixth year. At this period they will have through the gums four permanent grinders or molar teeth. Nothing is more common than for parents, as well as some physicians, to be greatly astonished when we tell them that these are their permanent teeth. These four molars are usually classed among the deciduous or first teeth; hence no effort is made to save them. We never fail to convince our patrons of their error when they bring their children to our office for examination. These molars frequently make their appearance before the child loses its two lower central incisors or front teeth, notwithstanding Dr. Dewees, in his work published in 1829, on diseases of children, says, "The permanent grinders do not usually appear until about the twelfth year." This is simply a mistake which dental research has corrected. About this time, between the fifth and sixth year, the lower central incisors drop out, and the permanent teeth come peering through the gums.

The lateral incisors make their appearance between the seventh and ninth; the first bicuspides between the ninth and tenth; the second between the tenth and eleventh; the cuspidati or eye teeth between the eleventh and twelfth; the second molars between the twelfth and fourteenth; the dentis sapientiæ, or wisdom teeth, between the eighteenth and twenty-second.

The temporary teeth ought to be frequently examined, and whenever decay makes its appearance, as we have already intimated, the decay should be arrested by filling or separating. Many reasons could be given for this decision which parents do not understand, but which we are always ready to explain. Suffice it to say that the durability, as well as the beauty of the permanent teeth very much depend upon the health of the first or temporary set. From the fifth to the fourteenth year of a child's life, if proper attention is given to the eruption and growth of the permanent teeth, the child may have in its mouth a beautiful and symmetrical arch, which, with care, may last through life; but if neglected, the beauty of the features, in all probability, will be seriously marred by projecting and irregular teeth, with the final loss of these important organs. Can we, then, too strongly impress upon the minds of those who have the care of children the importance of securing the services of some dentist who has been long enough engaged in the profession to give him experience in this department of science? We think not.

Go, then, with your children to the dentist of your choice, and if he understands his business you will receive instruction and services worth more to you and your children, by far, than all the cost and trouble connected with it. By this course you almost insure them good teeth in after life, a boon to them worth more than the gold of ophir. H. MARSHALL, Dentist.

